# PpSP42 and LmSTI1-PpSP42 Recombinant Proteins from *Phlebotomus papatasi* and *Leishmania major* Confer Protection Against *Leishmania major* Infection in BALB/c Mice

**DOI:** 10.34172/apb.45925

**Published:** 2026-05-09

**Authors:** Fariborz Bahrami, Touraj Miandoabi, Vahideh Moein Vaziri, Farhad Riazi-Rad, Soheila Ajdary

**Affiliations:** ^1^Department of Immunology, Pasteur Institute of Iran, Tehran, Iran; ^2^Department of Parasitology and Mycology, Shahid Beheshti University of Medical Sciences, Tehran, Iran

**Keywords:** Cutaneous leishmaniasis, Sand fly saliva, Leishmania major, LmSTI1, Immunogenicity, BALB/c mice

## Abstract

**Purpose:**

Cutaneous leishmaniasis (CL), a parasitic skin disease transmitted by sand flies, is characterized by complex interactions between *Leishmania* parasites and sand fly saliva, influencing both transmission and disease progression. This study investigated the immunogenicity of partial *Phlebotomus papatasi* salivary protein PpSP42 (or PP42 in short) and a novel recombinant fusion protein composed of *Leishmania major* stress-inducible protein 1 (LmSTI1) and PP42, produced in *Escherichia coli*.

**Methods:**

BALB/c mice were immunized subcutaneously with the fusion protein or with PP42 subunits separately, using resiquimod as an adjuvant, and subsequently challenged with *L. major* promastigotes and sand fly salivary gland homogenate. Lesion development and parasite burden as well as immune responses were evaluated by measuring key cytokines (IFN-γ, IL-4, IL-10, TNF-α) and antibodies (IgG, IgG1, IgG2a) up to eight weeks post-challenge.

**Results:**

Immunization with either PP42 or the fusion protein significantly attenuated lesion development (30-40% reduction in lesion size after 8 weeks; *P*<0.001) and parasite burden (∼100-fold lower after 8 weeks; *P*<0.05) compared to the PBS control. This protection was accompanied by significantly higher levels of IFN-γ and TNF-α (*P*<0.05 for both). Also, an elevated IgG2a/IgG1 ratio (*P*<0.01), indicative of a Th1-biased immune response was observed when the fusion protein was used for immunization.

**Conclusion:**

These findings suggest while immunization with PP42 protein alone resulted in lesion control and reduced parasite load, compared to the control group, these effects were less pronounced than those observed in the fusion protein group. Hence, LmSTI1-PP42 fusion protein warrants further investigation as a potential vaccine candidate against *L. major* infection.

## Introduction

 Leishmaniases are a set of parasitic infectious diseases, propagated by a protozoan parasite of the *Leishmania (L.)* species, which can cause diverse outcomes ranging from localized skin lesions, known as Cutaneous Leishmaniasis (CL), to mucosal damage and sometimes deadly forms that infect visceral organs, hence known as Visceral Leishmaniasis (VL).^1^ Leishmaniases remain a global health concern, especially in the developing world, with significant morbidity and mortality rates, while the quest for an effective human vaccine persists. CL is endemic to regions located in North Africa, Middle East, India, Southern Europe, and Central and South America.^2^ The infections are transmitted through sand fly vectors, which carry the parasite to their vertebrate hosts, where they reside within mononuclear phagocytes, such as macrophages.^3^ During a blood meal taken from an infected vertebrate host, the parasite enters the sand fly’s digestive tract and undergoes a transformation from the intracellular amastigote form to the flagellated extracellular promastigote form. During subsequent blood meals, the parasites, along with the sand fly saliva, enter the skin of the vertebrate host.^3^ In the New World, the sand fly vector belongs to the *Lutzomyia* (*Lu.*) genus, such as *Lu. longipalpis*, the vector for *L. infantum* that causes VL. In the Old World, *Phlebotomus* (*Ph.*) species are common *Leishmania* vectors, such as *Ph. papatasi*, which is the main vector of CL-causing *L. major* in Iran.^4^

 The development of an effective *Leishmania *vaccine has been pursued for many years using diverse technological platforms—from live-attenuated parasites^5^ to recombinant subunits and nucleic acid-based candidates.^6^ In recent years, the emergence of RNA vaccine technology offers a potent and practical solution, capable of inducing robust T-cell immunity, though its efficacy is contingent on advanced delivery formulations, such as lipid nanoparticles, to ensure stability and enhance cellular uptake. Furthermore, clinical evaluation is hampered by the logistical and financial burdens of conventional field trials in endemic regions.^7^

 Several *Leishmania* antigens have been evaluated as recombinant proteins in experimental subunit vaccine studies.^8-10^ While some of these antigens have demonstrated partial efficacy in protecting against *Leishmania* infections in animal models, the outcomes have often been deemed unsatisfactory. In some instances, these antigens have not yielded favorable results in primate studies or have necessitated the use of adjuvants that are unsuitable for human consumption.^11,12^ A well-investigated antigen is *L. major* stress-inducible protein 1 or in short LmSTI1.^13,14^ LmSTI1 is a 62.1 kDa heat shock protein that is expressed at both the amastigote and the promastigote stages of the parasite, with heightened expression levels observed under heat shock conditions. Compared to other *Leishmania* proteins, LmSTI1 has emerged as an immunogenic antigen, capable of eliciting a CD4 ^+^ Th1 response in mice.^15^

 Evidence suggests that beyond its function in insect feeding, sand fly saliva plays a significant role in the transmission of *Leishmania* parasites.^16-19^ It has been shown that the inoculation of *Leishmania* parasites in conjunction with sand fly saliva enhances the pathogenicity of the parasite.^20^ Moreover, investigations on both humans and animals have revealed that proteins present in sand fly saliva can stimulate both humoral and cellular immunity in various vertebrates.^21^ In fact, antibodies against sand fly saliva have been identified in mice, dogs, and individuals residing in endemic regions.^22,23^ In addition to comprehensive studies on sand fly saliva, specific protein components of sand fly saliva have also been individually investigated.^24,25^ For instance, examination of sera from individuals residing in an endemic area of VL in Brazil has revealed the presence of antibodies against specific *Lu. longipalpis* salivary proteins, namely LJM17 and LJM11 which are indicative of the sand fly bites.^21^ Interestingly, in another experiment conducted on residents of a CL endemic region in Tunisia, antibodies against a recombinant PpSP32 (that acts similar to the native form of the protein found in *Ph. papatasi* saliva), could be detected by their sera.^26^ Later investigations pointed to the positive attributes of LJM11, a 42 kDa salivary protein found in the saliva of *Lu. longipalpis* with respect to CL vaccine studies. LJM11 belongs to the family of yellow proteins and lacks homology with mammalian proteins. It has been shown that recombinant LJM11 can stimulate antibody production in both dogs and humans.^27,28^ Furthermore, experiments using an animal model of CL have shown that immunization with LJM11 alone, and without any adjuvant, confers protection against *L. major* infection, transmitted by sand fly bites.^29^

 A comparative analysis of genome sequences of *Lu. longipalpis* LJM11 with the *Ph. papatasi* genome revealed significant homology with a 42-kDa salivary gland protein known as PpSP42, also referred to as PP42. Previously, we successfully expressed the PP42 protein in HEK-293T eukaryotic cells.^30^ Building on the positive outcomes of the LJM11 studies, we investigated here the efficacy of recombinant PP42 and LmSTI1-PP42 fusion protein, using a BALB/c murine model of CL. We then evaluated lesion development, parasite burden, and both cellular and humoral immune responses upon a challenge infection with *L. major* and sand fly SGH. These experiments evaluated the immunogenicity and protective efficacy of PP42 and the fusion protein as potential vaccine candidates against CL. The results also provided several important insights into the functionality of this experimental vaccine.

## Materials and methods

###  Animals

 Female BALB/c mice (4–6 weeks old) were obtained from the animal facility of the Production Complex of the Pasteur Institute of Iran (Karaj, Iran). Mice were group-housed (4–6 per cage) in standard plastic cages under a 12-h light/dark cycle at a controlled temperature of 24 ± 2 °C. They had *ad libitum* access to a standard balanced diet, and water, in accordance with the pertaining ethics committee (92/0201/10253).

###  Parasite Culture

 An Iranian strain of *L. major* (MRHO/IR/75/ER) was cultured, initially in NNN medium and subsequently in RPMI 1640 medium supplemented with 10% fetal calf serum (FCS; Biosera, South Korea), 100 μg/mL streptomycin, and 100 U/mL penicillin, known as the complete medium.

###  Amplification of LmSTI1and Partial PP42 Genes

 Genomic DNA was extracted from 2 × 10⁸ *L. major* (MRHO/IR/75/ER) promastigotes. The parasites were harvested, washed with PBS, and resuspended in 100 μl of lysis buffer containing 50 μg/ml proteinase K, followed by incubation at 42°C for 2 h. The DNA was then extracted using phenol:chloroform:isoamyl alcohol (25:24:1), precipitated with 1/10 volume of 3 M sodium acetate (pH 7.0) and 2.5 volumes of ethanol at -20°C for 8 h. After centrifugation at 10,000 × *g* for 10 min, the resulting pellet was air-dried and resuspended in 100 μl of Tris-EDTA buffer. The DNA was stored at 4°C until use. The concentration and purity of the extracted DNA were assessed using a NanoDrop 1000 spectrophotometer (Thermo Fisher Scientific, USA). *Ph. papatasi* genomic DNA, kindly provided by Professor Parviz Parvizi (Department of Parasitology, Pasteur Institute of Iran), was used for amplification of the partial *PP42* gene. *LmSTI1* and partial *PP42* genes were amplified from *L. major* and *Ph. papatasi* genomic DNA, respectively, using the primers listed in Table 1 and ExprimeTaq DNA polymerase (Genet Bio, South Korea) according to the manufacturer’s instructions.

**Table 1:** Primers used for amplification of *LmSTI1* and partial *PP42*. the underlined and italic segments depict the sites for the restriction enzymes (*i.e.*, *BamH*I, *Sac*I and *Xho*I, used for the cloning purposes.

**Primers **	**5’-3’ Sequence**
LMST_BAMF	GTCT*GGATCC*GACGCAACTGAGCTGAAGAACAAG
LMST_SACR	GTCA*GAGCTC*GTCTGACCAAAACGAATGATGCCAGC
PP42_SACF	GACT*GAGCTC*AGCTTACGATTCAGGAAATATTGTAC
PP42_XHOR	GTG*ACTCGA*GCATAATGTCTGTGCCAAAATTGAAG

###  Cloning of LmSTI1 and PP42 Genes into the Expression Vector

*LmSTI1* and *PP42* amplicons were initially TA-cloned into the pTZ57R/T vector (InsTAclone, Thermo Scientific, USA) and transformed into PEG-competent *Escherichia coli* Top10 cells. Plasmids containing the inserts were extracted (TIANprep Mini Plasmid Kit, TIANGEN, China), confirmed by double digestion with the appropriate restriction enzymes (*BamH*I and *Sac*I for LmSTI1, and *Sac*I and *Xho*I for *PP42*), and gel-extracted (GeneJET Gel Extraction Kit, Thermo Scientific, USA). The purified inserts were then subcloned into the pET21a expression vector (Novagen, USA), pre-digested with the corresponding restriction enzymes, using T4 DNA ligase (TIANGEN, China). These constructs (pET21a-LmSTI1 and pET21a-PP42) were transformed into PEG-competent *E. coli* BL21(DE3) cells and verified by nucleotide sequencing (GenFanavaran Co., Iran). To generate the LmSTI1-PP42 fusion construct, the confirmed pET21a-LmSTI1 and pET21a-PP42 plasmids were double-digested with *Xho*I and *Sac*I. The linearized fragments were ligated using T4 DNA ligase. The resulting pET21a-LmSTI1-PP42 construct was transformed into *E. coli* BL21(DE3) cells and verified by nucleotide sequencing as above.

###  Expression and Purification of the Recombinant Proteins

*E. coli* BL21(DE3) strains harboring the expression constructs for the fusion protein, and PP42 were cultured at 37°C with shaking at 200 rpm. Optimal protein expression conditions were determined by culturing evaluating a range of IPTG concentrations (0.2, 0.5, 1.0, 1.5, and 2.0 mM) and induction times (1, 2, 3, and 4 h) at OD₆₀₀ of 0.6. Based on the results, expression was induced with 2 mM IPTG for subsequent experiments, except for the post-induction times which were 2.5 h for PP42, and 4 h for the fusion protein. Recombinant proteins were purified under denaturing conditions (8 M urea) using Ni-NTA columns (QiaExpressionist, Qiagen, Germany) according to the manufacturer’s protocol. Due to unsuccessful purification of PP42 using Ni-NTA columns, the bacterial lysate was separated by SDS-PAGE alongside a pre-stained marker. The gel was negatively stained using a reverse-staining technique with imidazole and ZnCl_2._^31^ The 42 kDa band was then excised and electroeluted (BioRad Model 422) in SDS-free buffer.

 In all cases, protein identity and purity were confirmed by SDS-PAGE and Western blotting. Briefly, following denaturation by boiling in Laemmli buffer for 5 min, samples were separated by SDS-PAGE on a 10% polyacrylamide gel; the gel was then stained with Coomassie Blue and destained with a methanol-acetic acid solution. For Western blotting, proteins from a similarly run gel were transferred to a PVDF membrane using a semi-dry electroblotter (Pharmacia LKB, Sweden). The membrane was subsequently blocked with 1% BSA in PBS for 2 h at room temperature and then incubated, with a 1:5000 dilution of using a monoclonal anti-histidine antibody (Anti-6X His tag antibody (HRP) ab1187; Abcam) in 1% BSA/PBS overnight at 4°C. After 1-h incubation at room temperature, the membrane was washed twice with PBST and once with PBS before the target bands were visualized through a colorimetric reaction using 3,3’-Diaminobenzidine tetrahydrochloride (DAB) substrate.

 Following purification, proteins were dialyzed extensively against PBS using 10 kDa cut-off dialysis bags, with decreasing urea concentrations (4 M, 2 M, and 1 M in PBS) over 6 h, followed by overnight dialysis against 2 L of pure PBS at 4°C to remove the urea. Endotoxins were removed using High Capacity Endotoxin Removal Resin columns (Pierce, USA) according to the manufacturer’s instructions. For all the purified proteins, the endotoxin level in the final protein preparations was confirmed to be < 0.1 EU/mL, as measured by the Limulus Amebocyte Lysate (LAL) assay performed by the Research and Production Complex of the Pasteur Institute of Iran, Karaj, Iran). Protein concentrations were determined using the Bradford assay.^32^

###  Immunization and Challenge

 Mice were divided into three groups: Group 1 received LmSTI1-PP42 fusion protein (n = 15), Group 2 received PP42 protein alone (n = 15), Group 3 served as the negative control group which received PBS (n = 15). All mice were immunized subcutaneously at the base of the tail 3 times, at 15-day intervals. Each immunization consisted of 10 μg of the respective protein and 10 μg of resiquimod as an adjuvant. The negative control group received PBS only. Three weeks after the last immunization, 5 mice from each group were bled and sacrificed for further evaluations. The remaining animals were challenged by inoculation subcutaneously with 10^5^ stationary phase promastigotes of *L. major* parasites in 50 µl PBS in the right hind footpad, along with equivalent of half a pair of salivary gland homogenate (SGH) per mouse (Figure 1). Female *Ph. papatasi* sand flies were generously provided by Professor Petr Volf (Charles University, Prague, Czech Republic) via infravec2 grant agreement No 731060. The SGH was prepared as previously described.^33^

**Figure 1:**
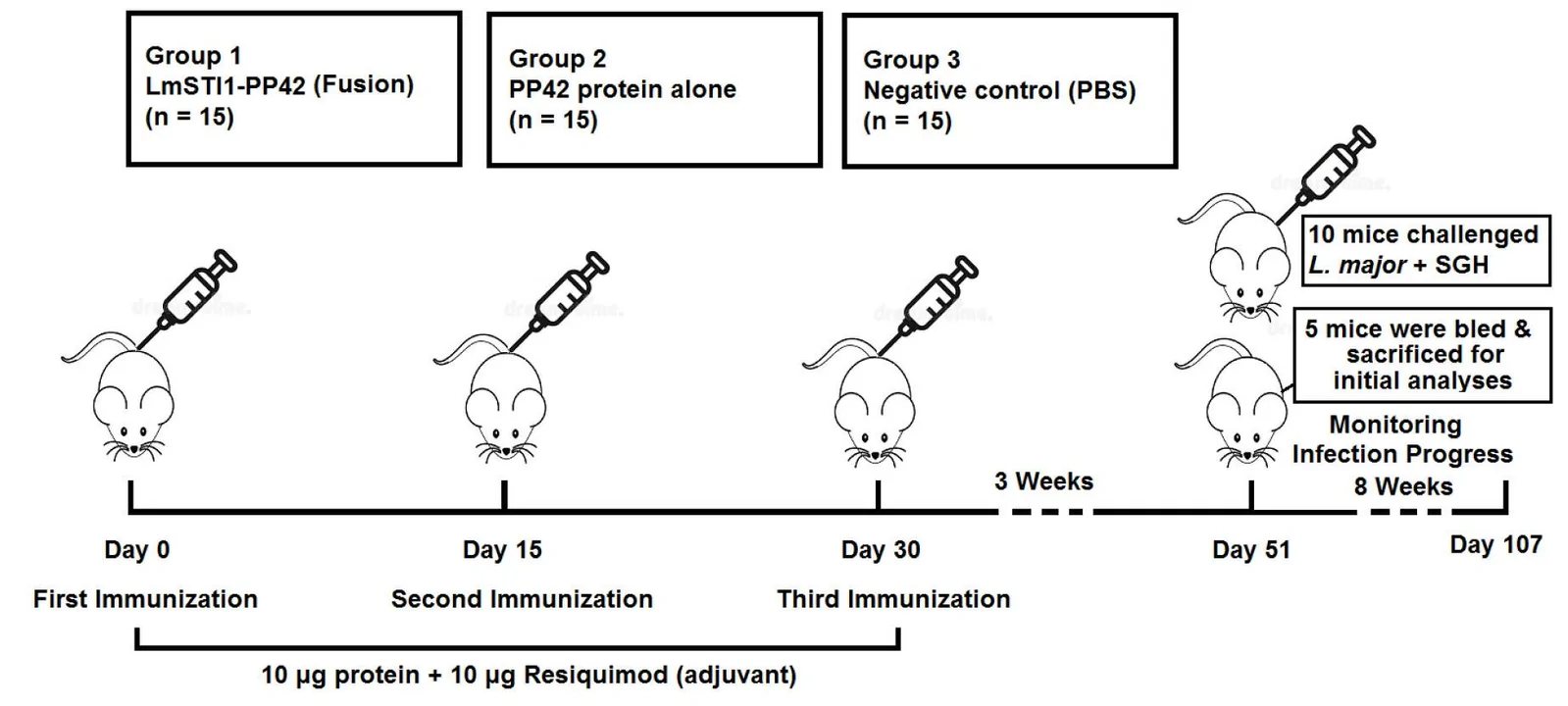
A timeline schematic of the mice groups, injections and analyses.As indicated, all BALB/c mice groups received three subcutaneous immunizations at the base of the tail at 15-day intervals. Three weeks after the final immunization, five mice per group were bled and sacrificed for the cytokine and antibody evaluations. The remaining animals (n = 10 per group) were challenged via subcutaneous inoculation in the right hind footpad with 10⁵ stationary phase *L. major* promastigotes plus SGH per mouse and monitored for the infection progress and immune response analyses till day 107 (*i.e.*, 8 weeks post-challenge) as indicated in the results

###  Assessment of Lesion Development and Parasite Burden 

 Footpad thickness was measured after the challenge weekly for eight weeks using a digital caliper (Mitutoyo, Japan). Lesion size was calculated as the difference in thickness between the infected and contralateral uninfected footpad. Parasite burden in the draining lymph nodes was determined in quadruplicate using a quantitative limiting dilution assay at four and eight weeks post-challenge, as previously specified.^34^ Briefly, popliteal lymph nodes were excised and homogenized by passing through a mesh stainless steel sieve into 5 mL of the complete medium, under sterile condition. Ten-fold serial dilutions of the homogenized tissue suspensions were then plated in 96-well plates and incubated at 24°C for 7 days. Plates were examined for viable and motile promastigotes by an inverted microscope, and the last dilution that was positive for motile parasites was considered as the final titer. Data analyzed by a method described by Titus and colleagues.^35^

###  Cytokine Assays 

 Three weeks after the final immunization (before the challenge) and at four and eight weeks following the challenge, spleen cells were harvested from each mouse (n = 5) and cultured according to previously established methods.^34^ Specifically, cell suspensions were prepared at a concentration of 2 x 10⁶ cells/mL. Each suspension was either left unstimulated (as a medium control) or stimulated with antigens: LmSTI1-PP42 fusion protein, PP42 alone, or *L. major* soluble leishmanial antigen (SLA). The cultures were then incubated for 4 days at 37°C in a humidified incubator with 5% CO₂. Following incubation, the supernatants were collected, and the levels of cytokines, namely IFN-γ, IL-4, IL-10, and TNF-α were quantified using a sandwich ELISA, with commercial kits (Mabtech, Sweden), and according to the manufacturer’s instructions.

###  Evaluation of Antigen-Specific Antibody Levels in Serum

 Serum samples collected 3 weeks after the final immunization (before the challenge), as well as four and eight weeks post-challenge were analyzed by indirect ELISA to determine levels of total IgG, IgG1, and IgG2a antibodies against SLA. ELISA plates (Greiner, Germany) were coated overnight with SLA and then blocked with PBS containing 1% bovine serum albumin (PBS-BSA). Serum samples, diluted 1:100 in PBS-BSA, were added to the wells and incubated for 1 h at 37°C. For total IgG detection, horseradish peroxidase (HRP)-conjugated goat anti-mouse IgG (Sigma-Aldrich, Germany) was added, followed by a 1-h incubation at 37°C and the addition of tetramethylbenzidine (TMB) substrate (Pishgamteb, Iran). For IgG subclass detection, goat anti-mouse IgG1 and IgG2a antibodies (Sigma-Aldrich, Germany) were added and incubated for 1 h at 37°C. Subsequently, HRP-conjugated rabbit anti-goat IgG (Sigma-Aldrich, Germany) was added, incubated for 1 h at 37°C, and TMB substrate was added. The reaction was stopped with 1 N HCl, and absorbance was measured at 450 nm using an ELISA plate reader (Anthos 2020, Austria).

###  Statistical Analyses

 Statistical analyses were performed using GraphPad Prism software version 8.0.2 (GraphPad Software Inc., 2019, San Diego, CA, USA). Comparisons between the immunized and control groups were performed using one-way ANOVA with Dunnett’s post hoc test. Data are presented as mean ± SD. A *P*-value of less than 0.05 was considered statistically significant.

## Results

###  Confirmation of Fusion Protein Expression and Purification

 The results confirm the successful amplification, cloning, and purification of the target proteins. Figure 2A shows the PCR amplification of the target genes using colony-touch PCR. Gel electrophoresis revealed distinct bands of the expected sizes (1645 bp for *LmSTI1* and 939 bp for *Pp42*) in colonies containing the pET21a expression vector, confirming successful amplification. *BamH*I digestion of the extracted pET21a-LmSTI1-Pp42 plasmid, followed by gel electrophoresis, yielded fragments of the expected sizes (5443 bp for pET21a vector and 2584 bp for *LmSTI1-Pp42* inserted fragment), demonstrating successful cloning and insertion of the target sequences (Figure 2B). SDS-PAGE analysis of purified LmSTI1-Pp42 protein revealed a band at approximately 100 kDa (Expected Mw: 99.3 kDa), indicating successful expression and purification of the recombinant fusion protein (Figure 2C). Western blot analysis further confirmed the presence of the target fusion protein at the expected molecular weight. These results demonstrated the successful cloning, expression, and purification of the LmSTI1-Pp42 fusion protein. The concentrations of the expressed proteins after the purification and urea-removal were evaluated to be 830 μg/ml for PP42, and 420 μg/ml for the fusion protein.

**Figure 2:**
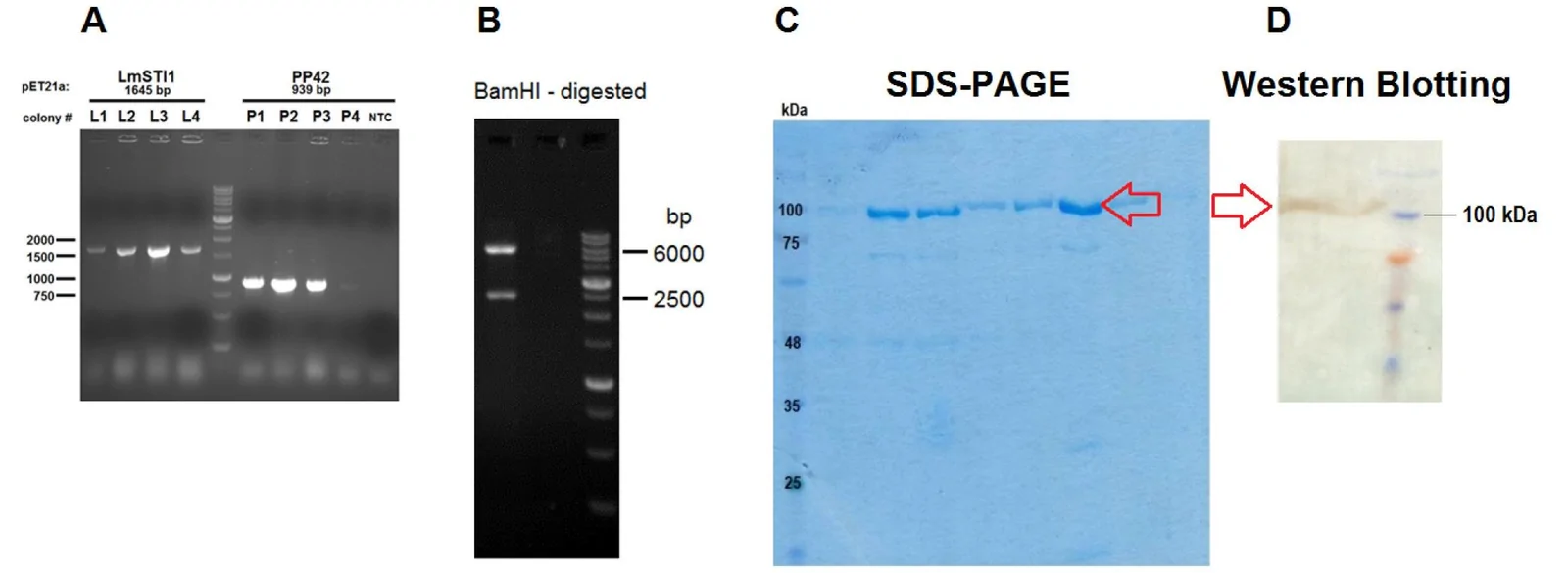
Cloning, expression, and purification of LmSTI1-PP42. **(A)** Colony-touch PCR amplification of *LmSTI1* (L1-L4) and *Pp42* (P1-P4) genes. Gel electrophoresis shows distinct bands of the expected sizes (*LmstI1*: 1645 bp; *Pp42*: 939 bp) in colonies containing the pET21a expression vector, confirming successful amplification. NTC: No template Control. **(B)***BamH*I digestion of the extracted *pET21a-LmSTI1-Pp42* plasmid. Gel electrophoresis of the digested fragments demonstrates the expected fragment sizes (5443 bp for pET21a vector and 2584 bp for *LmSTI1-Pp42* inserted fragment), confirming correct insert integration. **(C)** SDS-PAGE analysis of purified LmSTI1-Pp42 protein expressed in *E. coli* BL21. The gel shows a distinct band at approximately 100 kDa, corresponding to the expected molecular weight (99.3 kDa) of the recombinant fusion protein, indicating successful expression and purification. Molecular weight markers are included for reference. **(D)** Western blot analysis using an anti-His tag antibody confirmed the presence of LmSTI1-Pp42 at the same expected size, validating the expression and purification protocols

###  Reduced Lesion and Parasite Burden in Mice Immunized with LmSTI1-Pp42

 As depicted in Figure 3, immunization with the fusion protein resulted in reduced footpad swelling, as soon as three weeks following the challenge. The difference became increasingly prominent between the fifth and the eighth week post-challenge, compared to the control (*P* < 0.05). Notably, both the fusion protein and PP42 significantly decreased footpad swelling by the eighth week post-challenge (*P* < 0.001); however, the attenuation was more prominent when the fusion protein was used for immunization.

**Figure 3:**
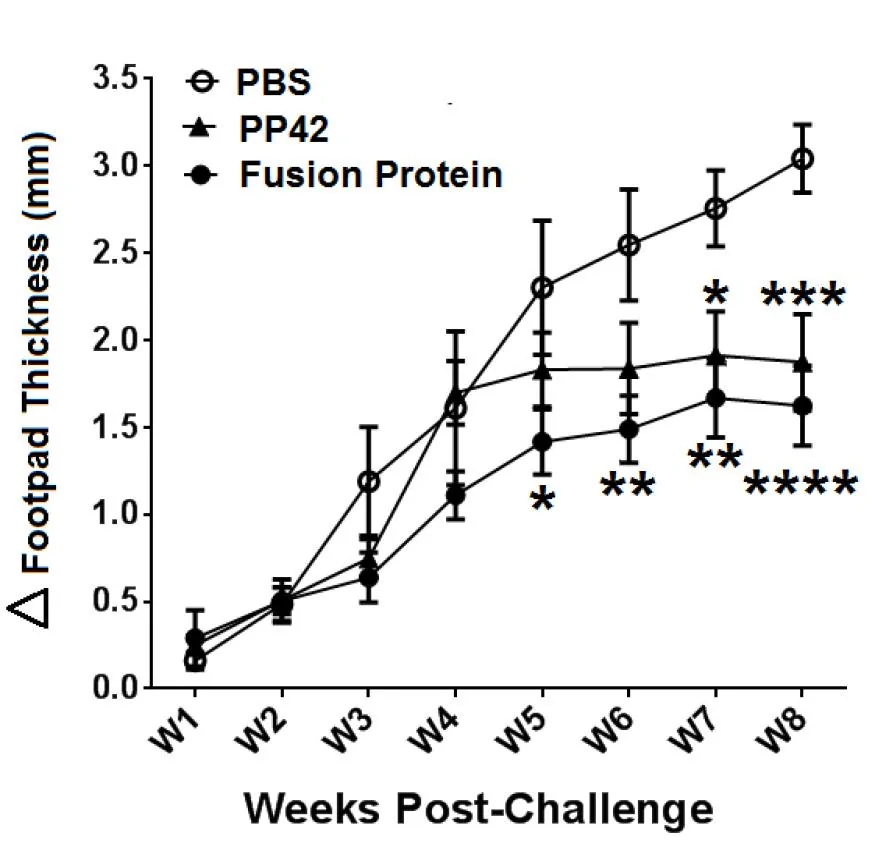
Footpad swelling (mm) over eight weeks post-challenge in mice immunized with the fusion protein, PP42 alone or PBS, and challenged with *L. major* promastigotes and SGH. Each point represents mean ± SD in swelling increase; when compared to the control group (PBS). *P < 0.05; **P < 0.01; ***P < 0.001; ****P< 0.0001

 Lymph node parasite burden was evaluated by limiting dilution assay at four- and eight-weeks post-challenge (Figure 4). Parasite burden per lymph nodes for the group immunized with the fusion protein was significantly reduced by approximately 2.5-fold at week four and over 400-fold at week eight post-challenge, compared to the control group (*P* < 0.05). The combined obtained data suggest the significant impact of the fusion protein on limiting the parasite propagation and the ensued lesion, compared to the groups immunized with PP42 protein alone or PBS, especially eight weeks after the challenge.

**Figure 4:**
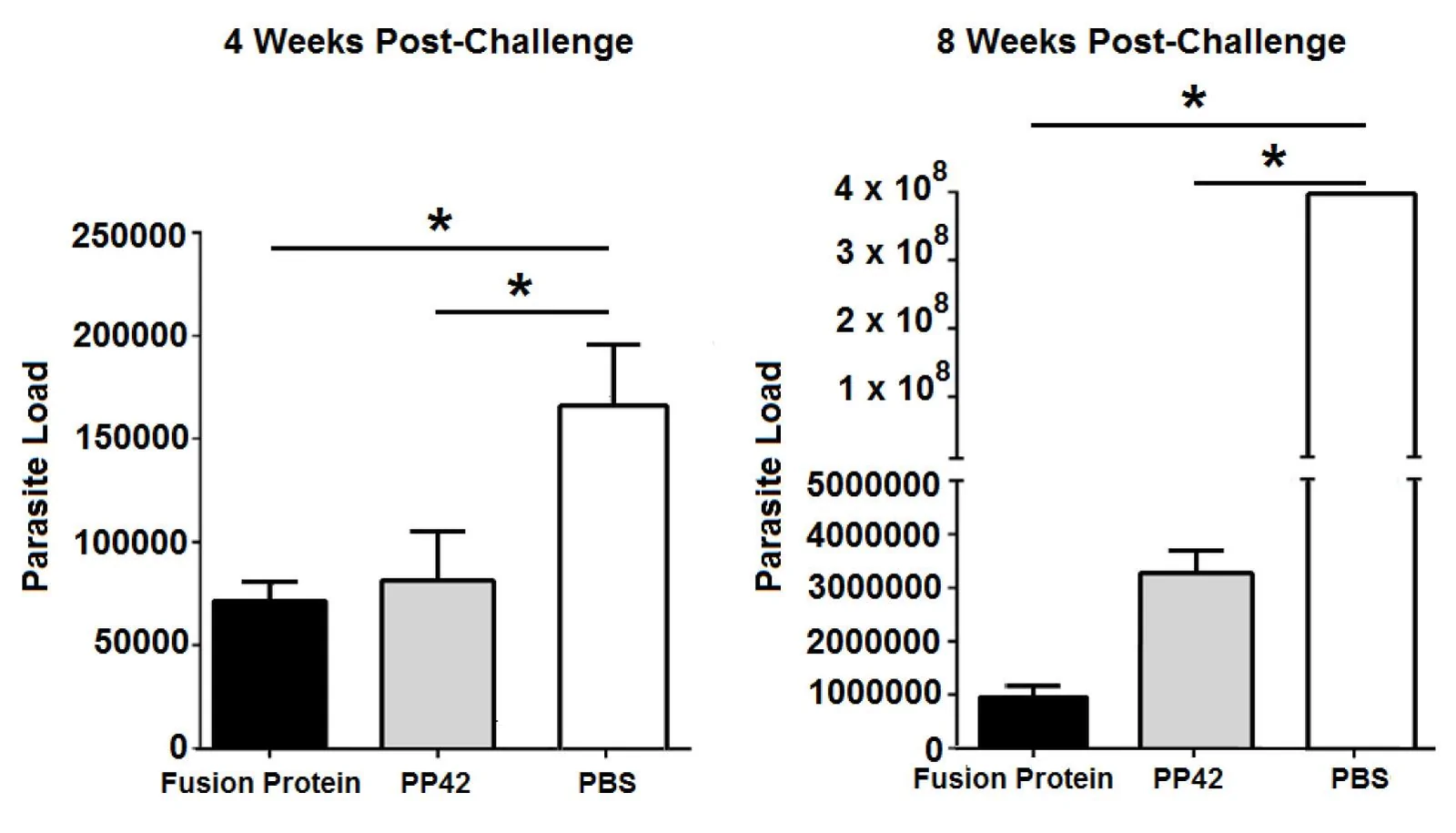
Parasite burden in draining lymph nodes at four weeks **(A)** and eight weeks **(B)** post-challenge, determined by limiting dilution assay in groups immunized with the fusion protein, PP42 alone or PBS, and challenged with *L. major* promastigotes and SGH. Results are mean ± SD n = 5 mice per group, compared with the PBS control group. Data are indicated by *P< 0.05

###  Evaluation of Antigen-Specific Cytokine Production by Splenocytes in the Immunized and Challenged Mice

 Based on a single-cell suspension from the spleen, cultured in the presence of the fusion protein, PP42 alone or SLA, the following results were obtained:

###  I. Significant IFN-γ Production due to Immunization with Fusion Protein

Figure 5A shows IFN-γ production by splenocytes stimulated with the fusion protein (left column), PP42 (middle column), or SLA (right column). Before challenge (top row), significant IFN-γ production was observed in mice immunized with fusion protein in response to the fusion protein and SLA (*P* < 0.05). Mice immunized with PP42 alone produced significant IFN-γ upon stimulation with PP42 (*P* < 0.01). Four weeks post-challenge (middle row), mice immunized with either the fusion protein or PP42 exhibited significantly elevated IFN-γ production upon stimulation with both recombinant proteins and SLA relative to the control group (*P* < 0.05). By eight weeks post-challenge (bottom row), mice immunized with the fusion protein showed significantly higher IFN-γ levels than the control when stimulated with the fusion protein, PP42, or SLA (*P* < 0.01). In contrast, the PP42-immunized group only produced significant IFN-γ in response to PP42 stimulation (*P* < 0.05).

**Figure 5:**
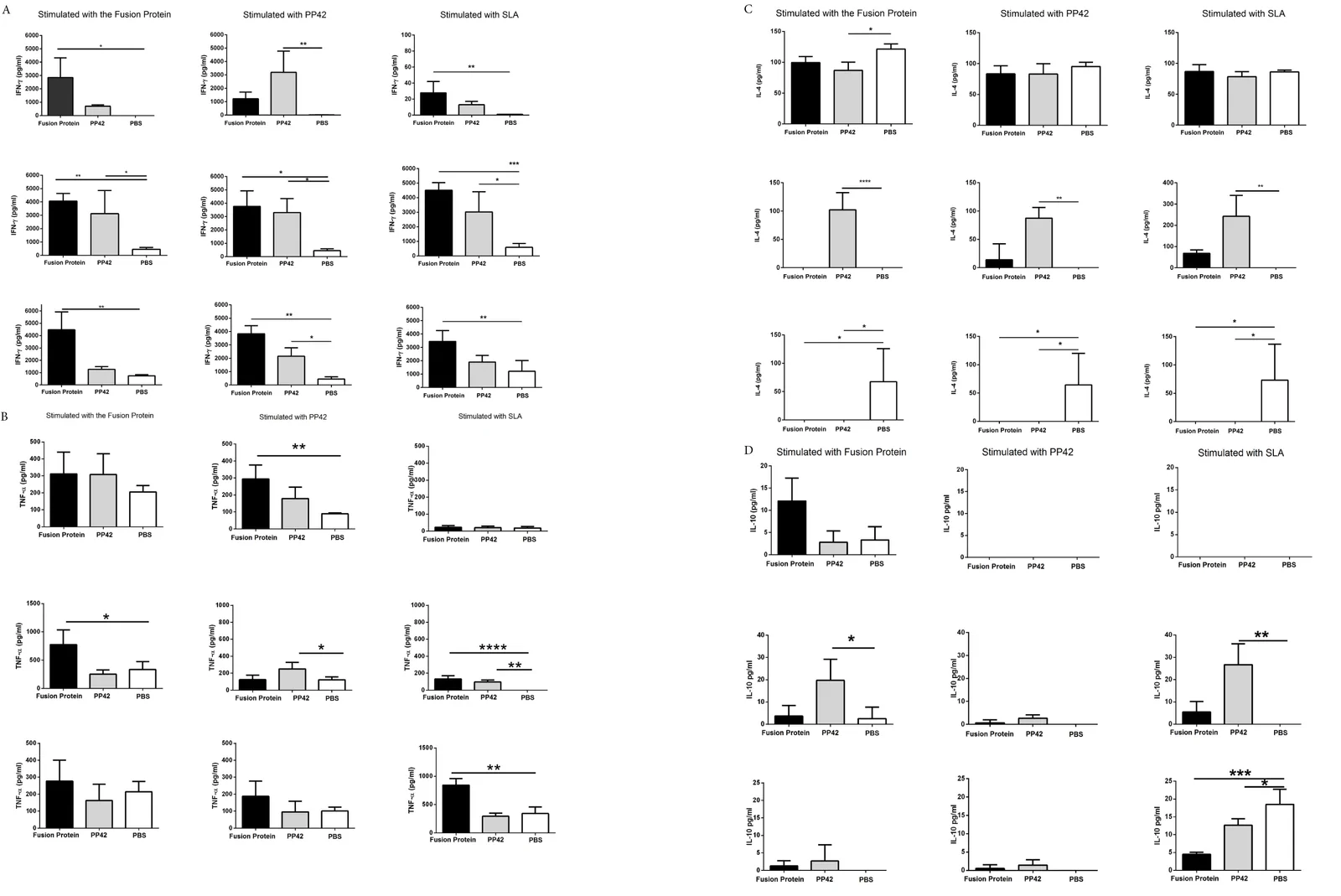
(A-D) Cytokine production in response to stimulation of the splenocytes with the fusion protein (left column), PP42 (middle column), or SLA (right column). Top row: Cytokine levels before challenge. Middle row: four weeks post-challenge. Bottom row: eight weeks post-challenge. Results are shown as the mean ± SD for five mice per group, compared with the PBS control group. Data are indicated by *P* < 0.05; *P* < 0.01; * *P* < 0.001; ***P < 0.0001.

###  II. Relatively high TNF-α Production Due to Immunization with the Fusion Protein 

 As shown in Figure 5B, prior to challenge (top row), mice immunized with the fusion protein showed significantly higher TNF-α production than controls when stimulated with PP42 (*P* < 0.01). At four weeks post-challenge (middle row), fusion protein-immunized mice produced significant TNF-α when stimulated with the fusion protein or SLA (*P* < 0.05). In addition, PP42-immunized mice showed significant TNF-α production in response to both PP42 and SLA (*P* < 0.05). By eight weeks post-challenge (bottom row), TNF-α production in fusion protein-immunized mice dropped sharply when stimulated with the fusion protein and remained low with PP42 stimulation. At this stage, significant TNF-α production in this group was observed only with SLA stimulation (*P* < 0.01).

###  III. Minimal IL-4 Production by Either the Fusion Protein or PP42 

Figure 5C depicts IL-4 production. Before challenge (top row), only the PP42-immunized group showed a marginally significant IL-4 response to the fusion protein (*P* < 0.05). At four weeks post-challenge (middle row), PP42 immunization alone induced significant IL-4 production in response to all three antigens (*P* < 0.01), while the fusion protein- immunized group exhibited negligible IL-4 responses. By week eight (bottom row), IL-4 production was further diminished in both the fusion- and PP42-immunized groups(*P* < 0.05).

###  IV. Low post-Challenge IL-10 Production in Mice Immunized with the Fusion Protein

 As shown in Figure 5D, before challenge (top row), only mice immunized with the fusion protein produced IL-10 upon stimulation exclusively with the fusion protein itself (*P* < 0.05); however, the difference compared to the control group was not statistically significant. All other groups and stimulation conditions showed negligible IL-10 levels. At four weeks post-challenge (middle row), PP42-immunized mice demonstrated a significant increase in IL-10 production in response to both fusion protein and SLA stimulation (*P* < 0.05). In contrast, the fusion protein-immunized group maintained minimal IL-10 production regardless of the stimulus. By eight weeks (bottom row), post-challenge, this group continued to show low to negligible IL-10 levels when stimulated with either the fusion protein or PP42 alone. Interestingly, the immunized groups showed significantly lower levels of IL-10 compared to the unimmunized group upon stimulation with SLA (*P* < 0.05).

###  Robust Th1-Related Antibody Response Elicited by Immunization with the Fusion Protein

 As indicated in Figure 6a, total serum IgG levels showed that prior to challenge, mice immunized with the fusion protein exhibited significantly higher total IgG compared to the control (*P* < 0.0001). The PP42 group did not produce notable antibody levels. At four and eight weeks post-challenge (Figures 6e and 6i), although IgG levels increased in all groups; no significant differences were observed among them.

**Figure 6:**
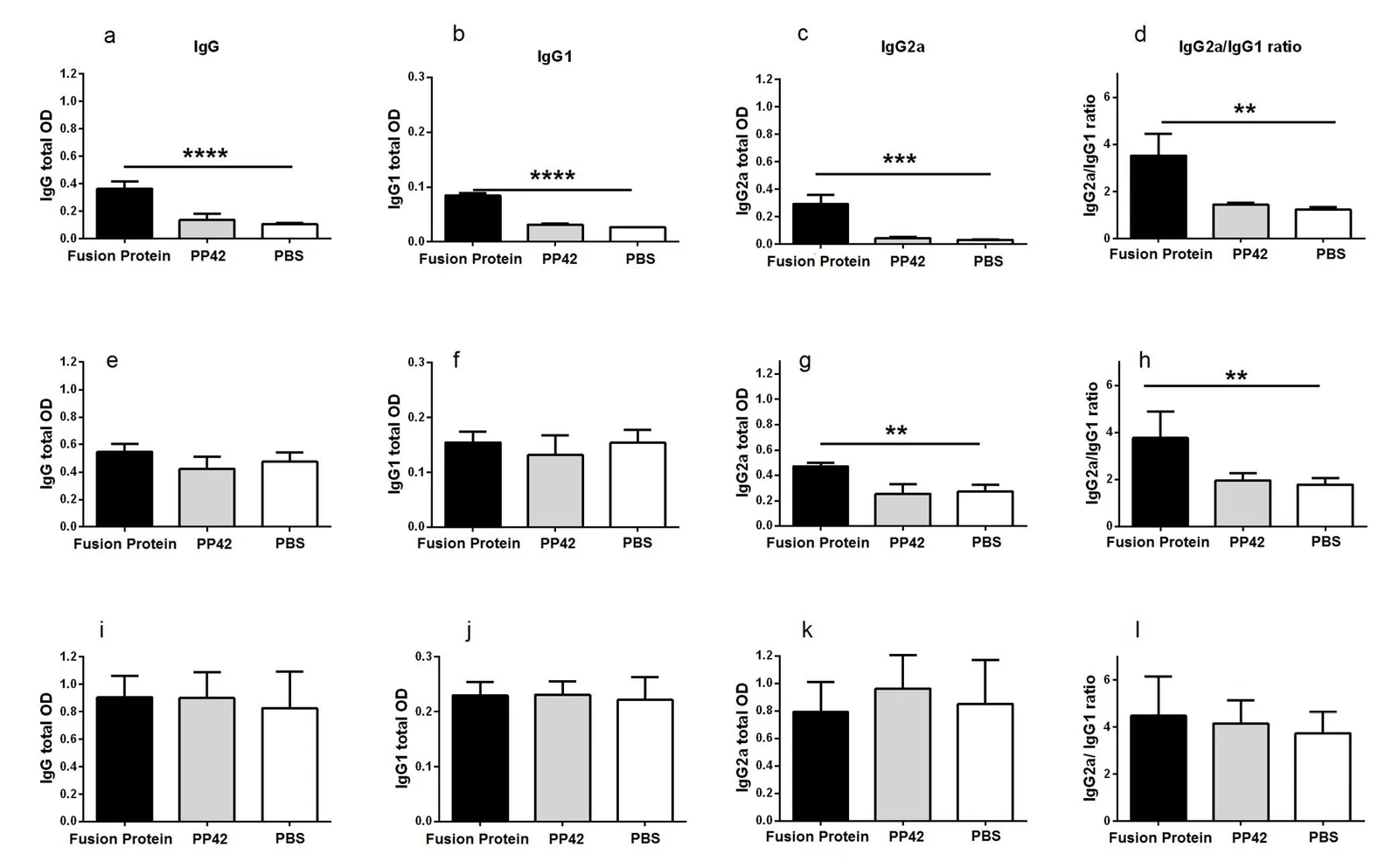
IgG, IgG1, and IgG2 antibody levels in immunized groups, three weeks after the final immunization (before challenge; a-d), as well as four (e-h) and eight (i-l) weeks post-challenge. Results are shown as the mean ± SD for five mice per group, compared with the PBS control group. Data are indicated by **P< 0.01; ***P< 0.001; ****P< 0.0001

 Our data revealed that before the challenge, mice immunized with the fusion protein had significantly higher IgG1 levels than the control (*P* < 0.0001), whereas the PP42 alone did not elicit a notable response (Figure 6b). Post-challenge, IgG1 levels across all groups showed no significant differences at both four and eight weeks, indicating that the initial IgG1 response was transient and not sustained long-term (Figures 6f and 6j). Moreover, prior to challenge, mice vaccinated with the fusion protein had significantly higher IgG2a levels than controls (Figure 6d; *P* < 0.01). This elevation persisted at four weeks post-challenge (Figure 6h; *P* < 0.01), suggesting a sustained Th1 response; however, by eight weeks, IgG2a levels across all groups converged (Figure 6l), implying the waning of this response over time. The PP42-only group induced increased IgG2a levels over time; however, it did not generate significant IgG2a antibody levels at any assessed time point.

## Discussion

 The failure to model natural transmission has been a major obstacle in developing effective anti-*Leishmania* vaccines. Many candidates that succeed in protecting against needle-injected parasites fail when challenged by infected sand fly bites.^36^ This critical discrepancy arises because a sand fly bite delivers not only parasites but also a complex cocktail of salivary proteins. Immunity against these salivary components has been shown to protect against disease, establishing them, as valuable vaccine targets.^37^ While promising, the use of native salivary gland homogenate is hindered by the need for sand fly colonies and laborious dissections. Consequently, the field has focused on producing recombinant salivary proteins. Immunization with recombinant LJM19protected hamsters from visceral leishmaniasis^21^, while PpSP15, salivary proteins from *P. papatasi* showed a significant reduction in lesion pathology, a decrease in parasite number, and the induction of *Leishmania*-specific immunity against *L. major *infection in mice and *Rhesus macaques.*^24,38^ The success of this strategy is further supported by modern approaches using fusion proteins (*e.g.*, PdSP15/LJL143), and confirms the critical importance of including salivary antigens in vaccine challenge models to predict real-world efficacy.

 LJM11 is an abundant yellow salivary protein and a member of the major protein family found in the saliva of sand flies from the genus *Lutzomyia.* Immunization of mice with LJM11 induced immunity and protection against *L. major* delivered by vector bites.^29^ By comparing *Lu. longipalpis* LJM11 with the *Ph. papatasi* genome, we previously found a significant homology with PpSP42 (PP42).^30^ For the first time in this study, we initially evaluated the immunogenicity and protective efficacy of PP42 in a murine model of CL following *L. major* along SGH challenge. Interestingly, mice immunized with PP42 controlled the infection and number of parasites in the lymph nodes compared to the non-immunized control group. The evaluation of cytokine results indicated that PP42 is immunogenic and induces significant levels of IFN-γ and IL-4 following *in vitro* stimulation of splenocytes with PP42. Meanwhile, no significant TNF-α and IL-10 were detected in pre-challenge mice. However, significant amounts of IFN-γ, TNF-α, IL-4 following stimulation with PP42 or SLA were released, post-challenge. Moreover, IL-10 levels were significant only in response to SLA. These results were in line with the previous results of immunization with homolog of this protein (*i.e.*, LJM11) which also induced IFN-γ, IL-10, and IL-4 and controlled the lesion size as well as parasite load, five weeks after an *L. major* challenge.^29^ The protection conferred by these salivary antigens is generally attributed to a saliva-specific, IFN-γ-driven Th1 response at the bite site. This rapid cellular reaction creates a hostile environment that indirectly impedes parasitic establishment^37,39^, positioning salivary proteins like PP42 as a potential vaccine candidate.

 The search for effective leishmaniasis vaccines has identified numerous parasite-derived antigens, capable of inducing protective immune responses.^6^ Among these, the conserved protein LmSTI1—present in both promastigote and amastigote forms—has shown significant promise in rodent models.^6^ Based on the complementary protective role of sand fly salivary antigens, we hypothesized that a fusion protein integrating LmSTI1 with salivary protein PP42 would provide superior protection. We therefore constructed this novel recombinant fusion protein, LmSTI1-PP42, and assessed its immunogenicity and protective efficacy against *L. major* challenge in a murine model of CL. It is noteworthy that the protective efficacy of the LmSTI1- PP42 fusion protein was initially evaluated against a non-covalent mixture of LmSTI1 plus PP42. The absence of a significant difference in protection (data not shown) led to the selection of the fusion protein (LmSTI1- PP42) for subsequent experiments, primarily because its production as a single polypeptide offers a substantial practical advantage.

 Monitoring footpad swelling revealed a marked reduction from weeks five through eight in mice immunized with the fusion protein, compared to the PBS control group, suggesting that the recombinant fusion protein can modulate the inflammatory response. This sustained control of inflammation could be crucial for managing CL symptoms, as excessive inflammation can exacerbate tissue damage.^40^ Consistent with previous studies^6,41^, the assessment of parasite burden in the lymph nodes revealed a significant decrease in the fusion protein group at four and eight weeks post-challenge, compared to the control group. The reduction was more prominent in the fusion group than in the PP42 group, suggesting a superior role for the fusion protein in limiting parasite replication and persistence.

 Immunization with the LmSTI1-PP42 fusion protein elicited robust cellular and humoral immune responses. Splenocytes from immunized and challenged mice, when stimulated with LmSTI1-PP42 or soluble *Leishmania* antigen (SLA), produced high levels of IFN-γ and TNF-α but little to no IL-4 or IL-10. The sustained induction of IFN-γ and TNF-α for up to eight weeks post-immunization, in response to multiple stimuli, indicated a durable Th1-type immune response. The synergistic action of IFN-γ and TNF-α is crucial for controlling *Leishmania* infection. As key mediators of the Th1 response, these cytokines activate macrophages to eliminate intracellular parasites through nitric oxide (NO) production. Consequently, IFN-γ is not only essential for parasite clearance but also strongly correlates with vaccine efficacy.^42^

 IL-4 promotes Th2 responses, which can counteract the protective effects of Th1 immunity by inhibiting macrophage activation. In *L. major* infection models, persistently high levels of IL-4 are associated with increased host susceptibility to disease.^43,44^ The fusion group exhibited minimal IL-4 throughout, indicating a skew away from Th2-type responses post-immunization and post-challenge. IL-10 plays a regulatory role in controlling inflammation to prevent excessive tissue damage. However, elevated IL-10 early in infection can suppress Th1 responses, allowing parasites to persist. Our data indicated that the fusion protein induces low levels of specific IL-10 response. While, four weeks post-challenge, the PP42-only immunized group shifted toward increased IL-10 production in response to multiple stimuli, potentially reflecting a regulatory or immunomodulatory response; the fusion protein elicited a predominantly Th1-biased immune profile with limited IL-10 expression as time progressed, highlighting its ability to promote cellular immunity while minimizing regulatory cytokine responses that could dampen protective immunity. This is considered favorable for pathogen control, given that IL-10 can suppress inflammatory responses and promote parasite survival.^45^

 Serum total IgG, IgG1, and IgG2a antibody levels were determined by ELISA. In murine vaccine models against CL, a moderate to high level of total IgG is desirable as it reflects broad B-cell activation and overall humoral immune engagement.^46^ While antibodies alone are insufficient for conferring protection—since cell-mediated immunity plays a predominant role—elevated IgG levels serve as an indicator of successful antigen recognition and effective immune system activation. Conversely, excessively high IgG levels in the absence of a Th1-skewed response may imply a non-protective or dysregulated immune profile.^47^ High IgG2a levels are considered a hallmark of protective, Th1-driven immunity. Driven by cytokines such as IFN-γ and TNF-α, IgG2a enhances macrophage-mediated pathogen clearance by opsonizing parasites for phagocytosis and supporting Fcγ receptor-mediated effector functions. In successful murine CL vaccine models, robust IgG2a responses correlate strongly with effective cell-mediated immunity, including macrophage nitric oxide production—an essential mechanism for intracellular pathogen killing.^46^ In murine models of CL, a high IgG2a-to-IgG1 ratio, significantly greater than 1, is indicative of Th1 polarization, reflecting the dominance of IFN-γ over IL-4. Such a ratio is commonly used as a molecular marker of vaccine efficacy, as a Th1-biased response enhances cellular immunity and pathogen clearance. Conversely, a low ratio (where IgG1 exceeds IgG2a) suggests a Th2 bias, which correlates with reduced vaccine effectiveness.^48^

 Before challenge, mice immunized with the fusion protein showed significantly higher total IgG, IgG1, and IgG2a levels compared to controls, indicating a robust, initial immune response with a Th1 bias (evidenced by an elevated IgG2a/IgG1 ratio). This Th1-skewed response persisted at four and eight weeks post-challenge. The PP42-only group did not produce notable antibody levels or shifts in IgG subclasses, suggesting it was less immunogenic on its own. The IgG2a predominance due to immunization with the fusion protein, indicative of a Th1 bias, aligns with the observed cellular immune response. This balanced antibody response can provide both immediate and long-term protection. Specifically, the IgG2a subclass is associated with complement activation and opsonization, critical processes for effective *Leishmania* clearance.^49^ In summary, while immunization with PP42 alone resulted in lesion control and reduced parasite load compared to the control group, these effects were less pronounced than those observed in the fusion protein group. Moreover, the fusion protein group exhibited more consistent and higher levels of IFN-γ and TNF-α, along with lower levels of IL-4 and IL-10, confirming a stronger induction of the protective immune response.

## Conclusion

 As a preclinical proof-of-concept, this study demonstrates that the novel recombinant fusion LmSTI1-PP42 protein, which integrates the *Leishmania* antigen LmSTI1 with the sand fly salivary protein PP42, represents a potential vaccine candidate against cutaneous leishmaniasis. The fusion strategy proved superior to immunization with the salivary component PP42 alone, yielding more effective control of lesion pathology and a significant reduction in parasite burden. This enhanced protection was driven by the induction of a robust and durable Th1-polarized immune response, Based on the current results, it is envisaged that the immunogenicity, protective efficacy and the long-term durability of the immune response due to this recombinant fusion protein should further be evaluated in more diverse and translationally relevant populations.

## Competing Interests

 There are no conflicts to declare.

## Ethical Approval

 All animal procedures were approved by the Ethics Committee of the Pasteur Institute of Iran (92/0201/10253) and conducted in accordance with relevant guidelines and regulations.
